# The circadian clock components BMAL1 and REV-ERBα regulate flavivirus replication

**DOI:** 10.1038/s41467-019-08299-7

**Published:** 2019-01-22

**Authors:** Xiaodong Zhuang, Andrea Magri, Michelle Hill, Alvina G. Lai, Abhinav Kumar, Srinivasa Bhargav Rambhatla, Claire L. Donald, Andrea F. Lopez-Clavijo, Simon Rudge, Katherine Pinnick, Wai Hoong Chang, Peter A. C. Wing, Ryan Brown, Ximing Qin, Peter Simmonds, Thomas F. Baumert, David Ray, Andrew Loudon, Peter Balfe, Michael Wakelam, Sam Butterworth, Alain Kohl, Catherine L. Jopling, Nicole Zitzmann, Jane A. McKeating

**Affiliations:** 1 Nuffield Department of Medicine, University of Oxford, Oxford OX3 7FZ, UK; 2 Oxford Glycobiology Institute, Department of Biochemistry, University of Oxford, Oxford OX1 3QU, UK; 3 Institute of Immunology and Immunotherapy, University of Birmingham, Birmingham B15 2TT, UK; 4 MRC-University of Glasgow Centre for Virus Research, University of Glasgow, Glasgow G61 1QH, UK; 5 The Babraham Institute, Cambridge CB22 3AT, UK; 6 Oxford Centre for Diabetes Endocrinology Metabolism, University of Oxford, Oxford OX3 9DU, UK; 7 Department of Chemistry, University of Birmingham, Birmingham B15 2TT, UK; 8 Institutes of Physical Science and Information Technology, Anhui University, Hefei 230601, China; 9 Inserm U1110, Institut de Recherche sur les Maladies Virales et Hépatiques, Strasbourg 67000, France; 10 Faculty of Medical and Human Sciences, University of Manchester, Manchester M13 9PL, UK; 11 Division of Pharmacy and Optometry, School of Health Sciences, Manchester Academic Health Sciences Centre, University of Manchester, Manchester M13 9NT, UK; 12 School of Pharmacy, University of Nottingham, Nottingham NG7 2RD, UK

## Abstract

The circadian clock regulates immune responses to microbes and affects pathogen replication, but the underlying molecular mechanisms are not well understood. Here we demonstrate that the circadian components BMAL1 and REV-ERBα influence several steps in the hepatitis C virus (HCV) life cycle, including particle entry into hepatocytes and RNA genome replication. Genetic knock out of *Bmal1* and over-expression or activation of REV-ERB with synthetic agonists inhibits the replication of HCV and the related flaviruses dengue and Zika via perturbation of lipid signaling pathways. This study highlights a role for the circadian clock component REV-ERBα in regulating flavivirus replication.

## Abstract (web summary)

The circadian clock can affect pathogen replication, but underlying molecular mechanisms are unclear. Here the authors demonstrate that the circadian components BMAL1 and REV-ERBα affect entry of hepatitis C virus (HCV) into hepatocytes and genome replication of HCV and related flaviviruses dengue and zika.

## Introduction

The cell-autonomous circadian clock coordinates the network of physiological processes that define the daily rhythms of cell proliferation, metabolism and inflammation^1^. Clock signalling pathways are primarily controlled by the transcription activators BMAL1 and CLOCK. The nuclear hormone receptors REV-ERBα and REV-ERBβ are BMAL1-regulated clock components that provide a feedback loop that controls the expression of metabolic genes in a circadian and tissue-dependent manner^2^. Host innate and adaptive immune response are now recognised to be circadian regulated and to influence susceptibility to infectious agents and response to vaccines^3–6^. Pariollaud et al. recently reported a homeostatic role for REV-ERBα in regulating pulmonary inflammation, coupling the core clock to innate immunity^7^.

As obligate intracellular parasites viruses require host cell machineries and metabolites to replicate their viral genome and to assemble progeny virions. Recent studies reporting increased replication of herpes, influenza^8^, respiratory syncytial virus and parainfluenza type 3^9^ in *Bmal1* knock-out mice suggest a role for circadian pathways to define host susceptibility to virus infection, however, the molecular mechanisms are not well understood. The recent availability of synthetic REV-ERB ligands that modulate circadian pathways in vivo^10,11^ provide tools to study the role of REV-ERB in viral replication and open exciting therapeutic opportunities for treating infectious disease.

The *Flaviviridae* family of positive-strand RNA viruses are major causes of morbidity and mortality and include the human pathogens: hepatitis C (HCV), dengue (DENV) and Zika (ZIKV) viruses. DENV infects around 390 million people per year^12^ and the recently emerged ZIKV has been estimated to infect 750,000 individuals annually since 2015^13^. To date, no anti-viral treatments are available for either virus. In contrast, the recent development of direct acting anti-viral agents (DAAs) against HCV infection has revolutionised treatment options^14^. However, given the high cost and limited availability of these drugs, significant numbers of chronic HCV-infected individuals remain at risk to develop progressive liver disease and hepatocellular carcinoma^15^. Despite differences in their transmission and pathogenesis, all of these viruses replicate in the cytoplasm and subvert lipid homeostatic pathways to induce organelle-like membrane structures that support viral replication^16^.

The liver maintains an organism’s metabolic homeostasis and REV-ERBα plays a key role in regulating bile acid and fatty acid biosynthesis^17–19^. As HCV replicates solely in hepatocytes within the liver and there are excellent in vitro model systems available to study its replication, we investigated the role of circadian clock components in the HCV life cycle. We show a circadian cycling of HCV entry into hepatocytes that is defined via BMAL1 regulation of entry receptors CD81 and claudin-1. Furthermore, we show that REV-ERB overexpression or activation with synthetic agonists inhibits HCV, DENV and ZIKV RNA replication, highlighting a new role for REV-ERB to restrict RNA virus replication.

## Results

### HCV infection is circadian regulated

**Fig. 1:**
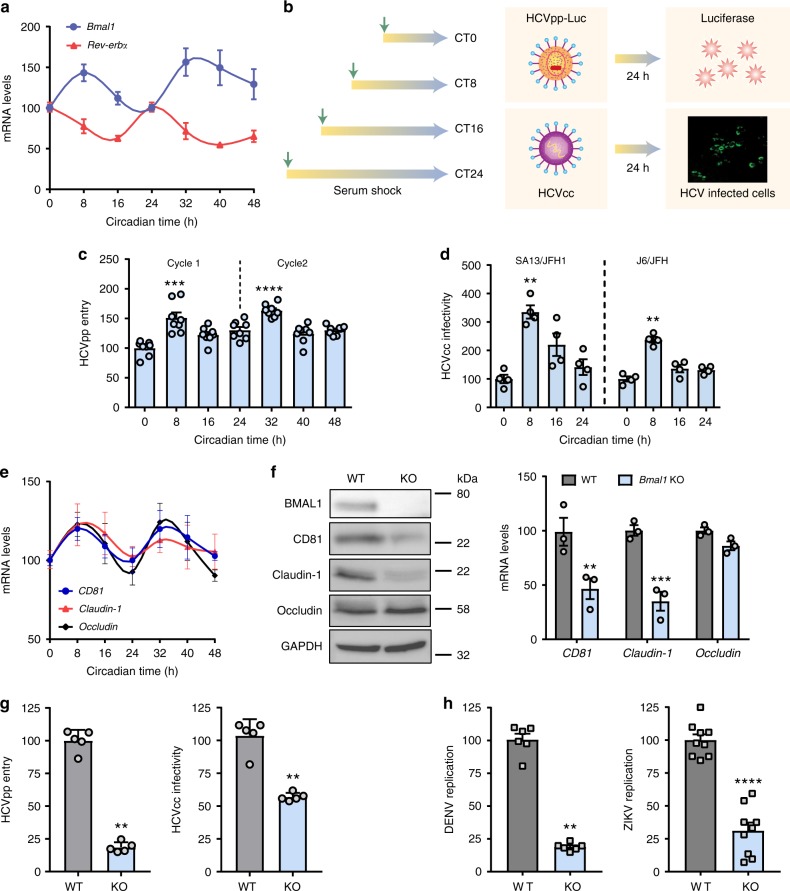
HCV entry is circadian regulated. **a** Synchronisation of Huh-7 cells. Huh-7 cells were serum shocked and *Bmal1* and *Rev-Erbα* mRNA measured  by quantitative reverse transcription polymerase chain reaction (qRT-PCR) and expressed relative to circadian time (CT) 0. Data are the average of four independent experiments (*n* = 4). **b** Circadian infection protocol. Synchronised Huh-7 were inoculated with HCVpp or HCVcc particles for 1 h and infectivity determined by luciferase assay or measuring the frequency of viral NS5A expressing cells 24 h later. **c** HCV entry shows a circadian pattern. Synchronised Huh-7 cells were inoculated with HCVpp at defined CTs and particle entry measured 24 h later and data expressed relative to CT0; mean ± S.E.M., *n* = 8, Kruskal–Wallis ANOVA with Dunn’s test. **d** HCV infection shows a circadian pattern. Synchronised Huh-7 were inoculated with HCVcc SA13/JFH-1 or J6/JFH-1 at defined CTs and the frequency of infected cells quantified 24 h later and data expressed relative to CT0; mean ± S.E.M., *n* = 4; Kruskal–Wallis ANOVA with Dunn’s test. **e** Circadian pattern of HCV receptors. Synchronised Huh-7 were lysed every 8 h, total RNA extracted and *CD81*, *claudin-1* and *occludin* mRNA levels together with the housekeeping *GAPDH* assessed by qPCR. Individual receptor transcripts were normalised to CT0. Representative of three independent experiments; *n* = 3, mean ± S.E.M. **f** BMAL1 regulates HCV entry receptors. Parental (WT) or *Bmal1* KO Huh-7 lysates were assessed for BMAL1 and viral receptors CD81, claudin-1 and occludin expression together with housekeeping GAPDH by western blotting. Total RNA from parental or *Bmal1* KO Huh-7 were extracted and mRNA of *CD81, claudin-1, occludin* and *GAPDH* measured by qRT-PCR. Data are expressed relative to parental cells (mean ± S.E.M., *n* = 3, Mann–Whitney test). **g** BMAL1 regulates HCV entry and infection. WT or *Bmal1* KO Huh-7 were inoculated for 1 h with HCVpp or HCVcc SA13/JFH-1 and infection assessed after 24 h. Data are expressed relative to WT cells (mean ± S.E.M., *n* = 5, Mann–Whitney test). **h** BMAL1 regulates DENV and ZIKV infection. WT or *Bmal1* KO Huh-7 were inoculated for 1 h with DENV or ZIKV and infection assessed after 24 h. Data are expressed relative to WT (mean ± S.E.M., *n* = 6 for DENV; *n* = 9 for ZIKV, Mann–Whitney test)

The human hepatocyte-derived cell line Huh-7 provides a well-characterised in vitro model to study the HCV life cycle and virus–hepatocyte interactions. Several approaches have been reported to synchronise the circadian clock in cell culture and we compared protocols that used dexamethasone, serum shock or temperature fluctuation to synchronise Huh-7 cells. Serum shocking the cells was the optimal protocol to coordinate the cycling of *Bmal1* and *Rev-Erbα* mRNA transcripts for 48 h (Fig. 1a), with the amplitude decreasing thereafter. Viral entry into a host cell represents the first step in the infectious life cycle and is mediated via specific interactions between virus proteins and cellular receptors that define particle internalisation pathways. Lentiviruses can incorporate exogenous viral encoded glycoproteins and the resulting pseudoparticles (pp) undergo a single cycle of infection that enable the study of receptor-specific internalisation pathways^20^. Synchronised Huh-7 cells at different circadian times (CTs) were inoculated with HCVpp for 1 h, unbound virus was removed by washing and infection was measured after 24 h (Fig. 1b). HCVpp entry was maximal at CT8 and CT32 (Fig. 1c). Delivering the lentiviral DNA directly into cells showed that serum-induced circadian synchronisation had no effect on reporter activity *per se* suggesting an HCV glycoprotein receptor-dependent pathway (Supplementary Figure 1A). Infecting synchronised cells at CT0 or CT8 with pseudotyped viruses expressing HCV or vesicular stomatitis virus (VSV) glycoproteins confirmed that CT8 cells support greater levels of HCVpp infection that was not apparent for VSVpp infection (Supplementary Figure 1B). The infectious HCV cell culture (HCVcc) system recapitulates the complete viral life cycle and we evaluated the infectivity of two well-characterised HCVcc strains, J6/JFH-1 and SA13/JFH-1, to infect Huh-7 cells at defined CTs. Infection was quantified by counting viral antigen NS5A expressing cells and we observed a significant increase in infected cell numbers when the virus was inoculated at CT8 (Fig. 1d), suggesting that circadian pathways regulate HCV uptake into the liver.

HCV entry into hepatocytes is regulated by four essential host factors: CD81, scavenger receptor BI (SR-BI), claudin-1 and occludin^21^. We investigated whether any of the viral receptors showed circadian expression and demonstrated a rhythmic pattern of CD81, claudin-1 and occludin mRNA levels in synchronised Huh-7 cells (Fig. 1e), with the peak of viral receptor transcripts coinciding with peak *Bmal1* mRNA levels and particle uptake. To establish a link between BMAL1 and receptor expression, we transiently silenced *Bmal1* in Huh-7 cells and observed a significant reduction in CD81 and claudin-1 transcripts (Supplementary Figure 2). CRISPR knockout (KO) of *Bmal1* in Huh-7 cells confirmed a significant reduction in CD81 and claudin-1 mRNA and protein levels, with no detectable change in occludin (Fig. 1f). Importantly, these *Bmal1* KO cells were resistant to HCVpp and HCVcc infection compared with wild-type (WT) cells (Fig. 1g). To ascertain whether BMAL1 regulates DENV or ZIKV infection, we infected the KO Huh-7 cells and observed a significant reduction in the replication of both viruses in *Bmal1* KO cells compared with WT (Fig. 1h). As the mechanism of particle entry for these viruses into hepatocytes is poorly defined and the tools to study viral uptake are not well developed, we selected to study the role of BMAL1 in HCV entry. In summary, these data support a role for BMAL1 to regulate HCV, DENV and ZIKV infection.

### Pharmacological activation of REV-ERB inhibits HCV entry

**Fig. 2:**
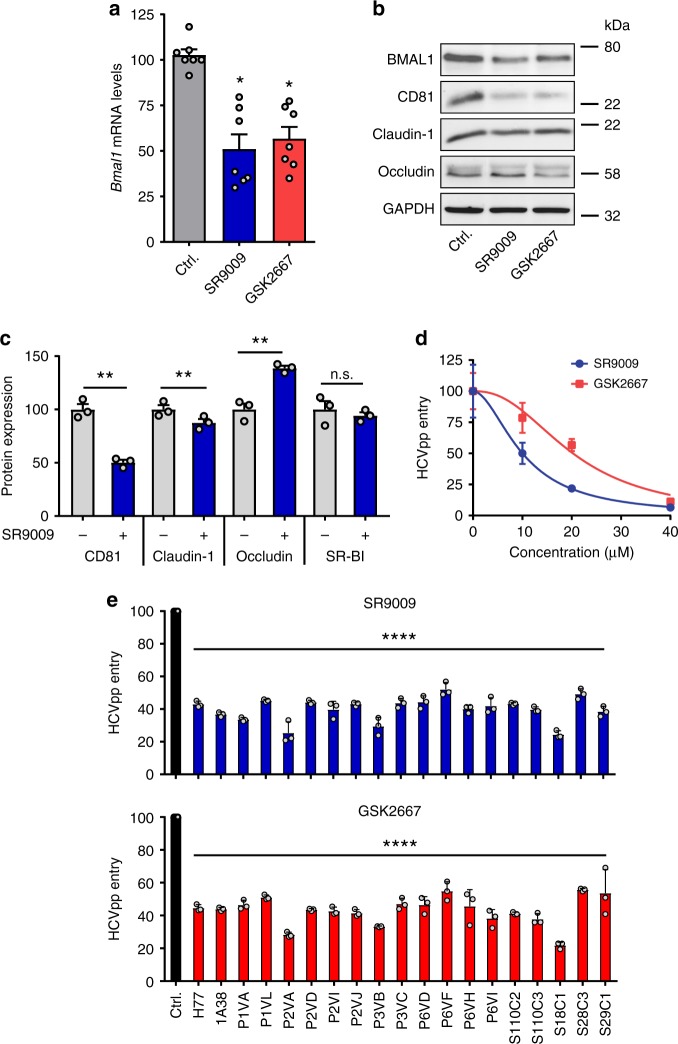
REV-ERB agonists inhibit hepatitis C virus (HCV) entry. **a** REV-ERB agonists inhibit BMAL1 transcription. Huh-7 cells were treated with REV-ERB agonists SR9009 or GSK2667 (20 µM) for 24 h and *Bmal1* mRNA levels quantified by qRT-PCR, respectively (mean ± S.E.M., *n* = 7, Kruskal–Wallis ANOVA with Dunn’s test). **b**, **c** REV-ERB agonists modulate HCV receptor expression. Huh-7 cells were treated with the REV-ERB agonists SR9009 or GSK2667 (20 µM) for 24 h and the cells lysed, total protein measured and assessed for CD81, claudin-1 and occludin expression together with the housekeeping GAPDH by western blotting or mass spectrometric analysis (mean ± S.E.M., *n* = 3, Mann–Whitney test). **d** REV-ERB agonists inhibit HCV entry. Huh-7 cells were treated with increasing dose of REV-ERB agonists SR9009 or GSK2667 for 24 h, inoculated with HCVpp and infection assessed 24 h later (mean ± S.E.M., *n* = 5). **e** REV-ERB agonists inhibit HCVpp bearing patient-derived glycoproteins. Huh-7 cells were treated with the REV-ERB agonists SR9009 or GSK2667 (20 µM) for 24 h, infected with HCVpp bearing patient-derived envelope glycoproteins and infection assessed 24 h later. In all cases, data are expressed relative to untreated (Ctrl) cells. (Mean ± S.E.M., *n* = 3, Kruskal–Wallis ANOVA)

*Bmal1* is negatively regulated at the transcriptional level by REV-ERBs, haem-binding transcriptional repressors^22^. The development of synthetic agonists that activate REV-ERB and modulate circadian pathways in vivo^10,11^ prompted us to investigate their ability to regulate HCV infection. REV-ERB agonists, SR9009 and GSK2667, reduced BMAL1 promoter activity in a dose-dependent manner (Supplementary Figure 3) and we confirmed a reduction in endogenous mRNA levels and protein expression (Figs. 2a, b) with no detectable cytotoxicity or effect on hepatocellular viability (Supplementary Figure 3). To exclude the possibility of global transcriptional repression by these agonists, we assessed the expression level of nine housekeeping genes in treated Huh-7 cells and found no significant changes in their mRNA levels (Supplementary Figure 4). REV-ERB agonists treated Huh-7 cells showed a significant reduction in CD81 expression assessed by western blotting with negligible effects on claudin-1 or occludin expression (Fig. 2b). As measuring protein expression by western blotting is semiquantitative and partly defined by affinity and avidity of the antibodies being used, we selected a non-biased proteomic approach to assess the effect of SR9009 on viral receptor expression. We observed a significant reduction in CD81 and claudin-1 expression with an increase in occludin levels and no significant change in SR-BI (Fig. 2c). Importantly, both REV-ERB agonists inhibited HCVpp entry in a dose-dependent manner (Fig. 2d). To evaluate the activity of these agonists against a wider spectrum of HCV strains, we utilised lentiviral pseudotypes expressing genetically diverse envelope glycoproteins and demonstrated inhibition of all strains tested (Fig. 2e). In summary, these data highlight a role for REV-ERB to regulate CD81 and claudin-1 protein expression and define circadian gating of HCV entry.

### REV-ERB limits HCV RNA replication

**Fig. 3:**
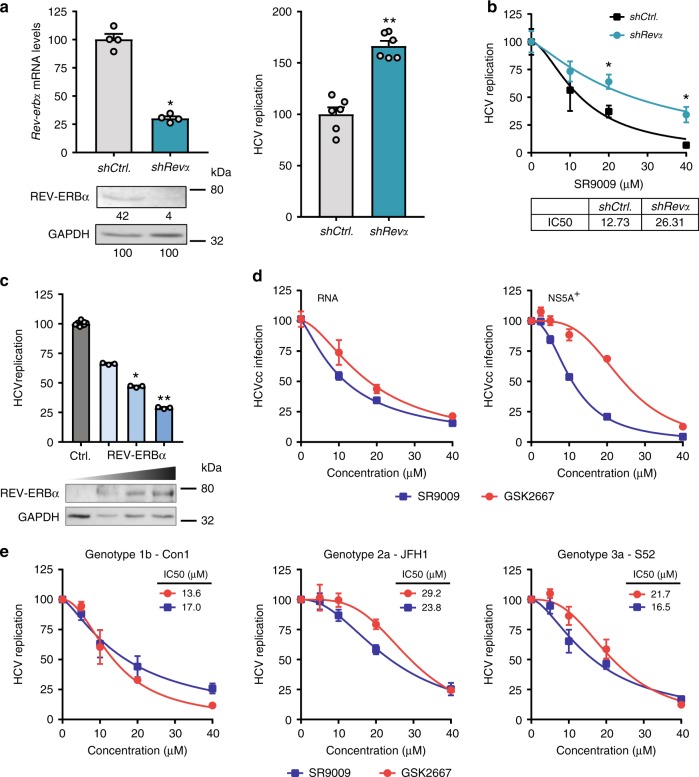
REV-ERBα inhibits hepatitis C virus (HCV) RNA replication. **a** Silencing Rev-erbα increases HCV replication. Huh-7 cells supporting a HCV JFH-1-LUC replicon were transduced with lentivirus encoding sh*Rev-erbα* or control and silencing confirmed by measuring *Rev-erbα* mRNA and protein expression levels (mean ± S.E.M., *n* = 4, Mann–Whitney test). Densitometric analysis quantified REV-ERB in individual samples and was normalised to its own GAPDH loading control. HCV replication-dependent reporter activity was measured and expressed relative to control (shCtrl) cells (mean ± S.E.M., *n* = 6, Mann–Whitney test). **b** Anti-viral activity of SR9009 agonist is dependent on REV-ERB expression levels. sh*Rev-eRbα* and Ctrl HCV JFH-1 replicon cells described in (**a**) were treated with REV-ERB agonist SR9009 for 24 h, viral replication measured and the concentration of agonist required to inhibit viral replication by 50% defined (IC_50_) (mean ± S.E.M., *n* = 3). **c** REV-ERBα overexpression inhibits HCV RNA replication. Huh-7 cells stably supporting a HCV JFH-1-LUC replicon were transfected with empty plasmid or vector expressing REV-ERBα and 48 h later protein expression assessed by western blotting and viral replication measured (mean ± S.E.M., *n* = 4, Mann–Whitney statistical test). Data are plotted relative to Ctrl untreated cells. **d** REV-ERB agonists cure HCV-infected cells. HCVcc SA13/JFH-1 infected Huh-7 cells were treated with increasing concentrations of REV-ERB agonists for 24 h and viral RNA or NS5A-expressing cells quantified and data expressed relative to Ctrl untreated cells. The experiment was performed in the presence of a neutralising anti-CD81 antibody to limit secondary rounds of infection (mean ± S.E.M., *n* = 3). **e** REV-ERB ligands inhibit the replication of diverse HCV genotypes. Huh-7 cells transiently supporting HCV sub-genomic replicons representing genotypes 1–3 were treated with the REV-ERB agonists SR9009 or GSK2667 and replication assessed 24 h later. The dose of agonist required to inhibit HCV RNA replication by 50% (IC_50_) was determined for all viral genotypes (mean ± S.E.M., *n* = 3)

HCV can establish a persistent infection and sub-genomic copies of the viral RNA can replicate autonomously. These replicon systems have been widely used as pre-clinical models for anti-viral drug discovery. Short hairpin RNA silencing *Rev-erbα* in Huh-7 cells stably supporting HCV-luciferase replicon increased viral replication (Figs. 3a, b), demonstrating a role for REV-ERB to regulate viral genome replication after particle entry. Furthermore, treating HCV replicon cells with REV-ERB agonist SR9009 reduced viral replication in a dose-dependent manner and efficacy was significantly reduced in the sh*Rev-erbα* cells compared with control cells (Fig. 3b), confirming the anti-viral activity of SR9009 is dependent on REV-ERB protein levels. We noted that the endogenous expression of REV-ERBα in Huh-7 cells was low (Fig. 3a) and hypothesised that overexpression would limit HCV replication. Transient expression of REV-ERBα in HCV replicon cells inhibited viral genome replication in a dose-dependent manner (Fig. 3c). Given these promising data, we assessed the effect of REV-ERB agonists to modulate genome replication in HCVcc-infected cells, where a neutralising anti-CD81 receptor antibody was included to block secondary infection events^23^. We observed a dose-dependent reduction in HCV RNA levels and NS5A-expressing cells with both agonists (Fig. 3d). Transient delivery of HCV RNA into *Bmal1* KO cells bypassed the restriction in viral receptor-dependent particle entry and enabled us to show that REV-ERB agonist SR9009 inhibits HCV replication in WT and *Bmal1* KO cells (Supplementary Figure 5), showing a role for REV-ERB to regulate HCV RNA replication independent of *Bmal1* expression. Finally, we demonstrate that both REV-ERB agonists inhibit the replication of diverse HCV genotypes 1b (Con1), 2a (JFH-1) and 3a (S52)^24^ (Fig. 3e).

### REV-ERB regulates miR-122

**Fig. 4:**
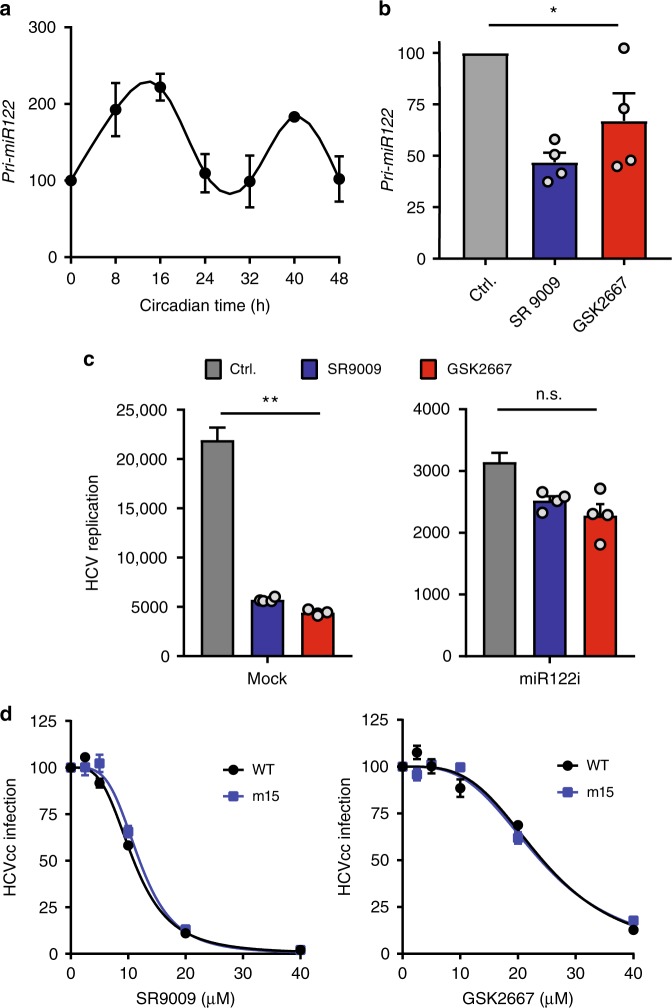
REV-ERB agonists modulate miR-122. **a** Pri-miR122 displays circadian pattern in synchronised Huh-7 cells. qRT-PCR analysis of pri-miR-122 levels relative to GAPDH in cycling Huh-7 cells (mean ± S.E.M., *n* = 3). **b** REV-ERB agonists reduce pri-miR122 levels. qRT-PCR analysis of *pri-miR-122* relative to GAPDH in Huh-7 cells treated with REV-ERB agonists (20 μM) for 16 h (mean ± S.E.M., *n* = 3, Kruskal–Wallis ANOVA with Dunn’s test). **c** Anti-viral activity of REV-ERB agonists is miR-122 dependent. Huh-7 cells were electroporated with HCV JFH-Luc and miR-122 antagonist (100 pM per 10^6^ cells) or a randomised control, allowed to recover for 4 h and treated with REV-ERB agonists (20 μM) for 24 h. Hepatitis C virus (HCV) replication was assessed by measuring luciferase activity and the data expressed relative to Ctrl untreated cells (Mean ± S.E.M., *n* = 4, Kruskal–Wallis ANOVA with Dunn’s test). **d** REV-ERB agonists inhibit HCV independent of direct miR-122 binding. Huh-7 cells were inoculated with WT or miR122 independent HCV (m15) for 6 h, unbound virus removed by washing and the infected cells treated with SR9009 or GSK2667 for 48 h and the frequency of NS5A expressing cells quantified and expressed relative to untreated (Ctrl) cells (mean ± S.E.M., *n* = 3)

HCV RNA binds the liver-specific microRNA miR-122 and this complex has been reported to support the replication of the viral RNA^25^. As REV-ERBα regulates miR-122 and both primary (pri)-miR-122 transcription and miR-122 target genes show circadian patterns of expression^26^, we were interested to investigate the miR-122 dependency of REV-ERB restriction of HCV replication. Synchronised Huh-7 cells show a circadian pattern of pri-miR-122 expression (Fig. 4a) and REV-ERB agonists reduced pri-miR-122 levels (Fig. 4b). Co-transfection of a miR-122 antagonist along with HCV RNA into Huh-7 cells reduced virus replication as previously reported^25^ and both REV-ERB agonists showed reduced anti-viral activity (Fig. 4c), suggesting a role for REV-ERB regulation of miR-122 in restricting HCV replication. To investigate further, we evaluated the sensitivity of a HCV variant encoding a mutated miR-122 binding motif that recognised miR-15a/b^27^ (m15) to REV-ERB agonists. Both SR9009 and GSK2667 inhibited m15 replication with comparable efficiency as WT virus (Fig. 4d), demonstrating that REV-ERB agonists inhibit HCV replication independent of miR-122 binding to the viral RNA. As miR-122 regulates cholesterol and lipid metabolism^28^, this most likely contributes to the anti-viral activity of REV-ERB agonists to limit HCV replication.

### REV-ERBα regulates pathways essential for HCV, DENV and ZIKV

REV-ERBα regulates lipid and cholesterol metabolism in the murine liver^19,29^. As lipids play a role in the genesis and maintenance of membranous vesicles that are essential for RNA virus replication and particle assembly^16^, we investigated the effect of the REV-ERB agonists on de novo lipogenic pathways in human hepatocytes. As SR9009 showed the highest anti-viral activity and has been extensively studied in vivo, we performed a whole-genome microarray on SR9009 (20 µM) treated Huh-7 cells.

**Fig. 5:**
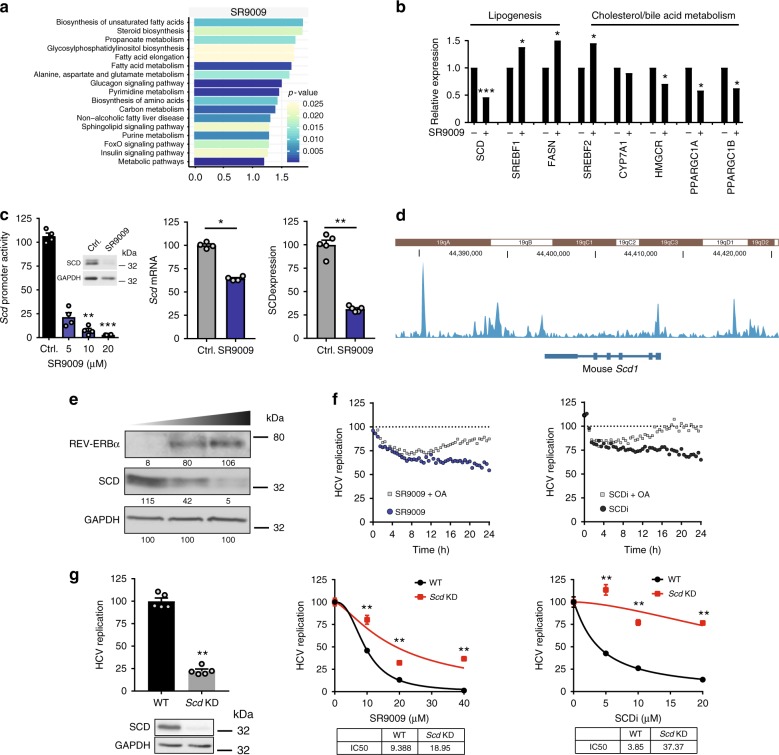
REV-ERB activation perturbs fatty acid metabolism. **a** REV-ERB agonist SR9009 perturbs metabolic pathways. Huh-7 cells were treated with SR9009 (20 µM) or vehicle control for 24 h and gene expression analysed by human genome microarray (biological replicates n = 3). Differentially expressed genes were assessed by KEGG pathway enrichment analysis. **b** Metabolic genes perturbed by SR9009. Relative expression of metabolic genes involved in lipogenesis, cholesterol and bile acid metabolism following SR9009 (20 µM) treatment. **c** REV-ERB agonist inhibits SCD promoter activity and protein expression. Huh-7 cells were treated with REV-ERB agonist SR9009 (20 µM) for 24 h and SCD promoter activity (mean ± S.E.M., *n* = 4, Kruskal–Wallis ANOVA with Dunn’s test), transcript levels and protein expression assessed by western blotting and mass spectrometric analysis. **d** REV-ERBs bind SCD1 promoter. Analyses of available ChIP-seq data in mouse livers^30^ demonstrate REV-ERB peaks in SCD1 promoter. Read densities for REV-ERBα tracks are represented by height on the *y* axis. **e** Overexpressing REV-ERBα inhibits SCD expression. HCV A2-Luc replicon cells were transfected with an increasing dose of REV-ERBα expression plasmid. Cell lysates were collected 48 h post-transfection and assessed for SCD expression, together with housekeeping GAPDH by western blotting. **f** Oleic acid partially restores anti-viral activity of REV-ERB agonist SR9009. HCV replicon cells were treated with SR9009 at 15 µM (left) or SCD inhibitor A939572 (SCDi) at 10 µM (right) alone or in combination with oleic acid (OA) at 100 µM. HCV RNA replication was monitored at 30 min intervals for 24 h. **g** A role for SCD in REV-ERB agonist inhibition of HCV replication. HCV replicon cells were transfected with CRISPRs targeting exons 2 and 3 of SCD or a scrambled guide RNA and 24 h later treated with SR9009 or SCD inhibitor A939572. SCD expression was assessed by western blotting and the dose of REV-ERB agonist or SCD inhibitor required to inhibit HCV RNA replication by 50% (IC_50_) in control or knock-down (KD) cells determined (mean ± S.E.M., *n* = 5, Mann–Whitney statistical test)

Differentially expressed genes from the microarray data were filtered through the following criteria: Log2 fold change magnitude > 0.5, a *p*-value < 0.05, which generated a list comprising 4033 upregulated genes and 3660 downregulated genes (most highly regulated genes are listed in Supplementary Data 1). KEGG pathway analysis of the differentially expressed genes identified an enrichment of metabolic pathways involved in lipogenesis and cholesterol/bile acid metabolism (Figs. 5a, b). We demonstrated that SR9009 reduced stearoyl-CoA-desaturase (SCD) promoter activity, mRNA transcript levels and protein expression assessed by western blotting and proteomics analysis (Fig. 5c). Furthermore, SR9009-treated Huh-7 cells showed a significant reduction in unsaturated fatty acid levels (16:1/16:0—ctrl 0.42v SR9009 0.38, *p =* 0.0018; 16:1 + 18:1n-7—ctrl 20.41v SR9009 18.98, *p =* 0.0001; 16:1 + 18:1n-7/16:0—ctrl 0.77v SR9009 0.72, *p =* 0.0065), a phenotype consistent with reduced SCD expression and activity. Trump et al. reported that REV-ERB agonists activate the nuclear receptor LXRα in human monocyte THP1 cells^10^. As LXR regulates metabolic pathways that overlap with REV-ERBα, we evaluated the ability of SR9009 to activate LXR and demonstrated a negligible effect on known LXR target gene transcription in human hepatocytes (Supplementary Figure 6). Analysis of published ChIP-seq data^30^ on REV-ERB chromatin occupancy in murine liver showed evidence of binding the SCD1 promoter (Fig. 5d). Overexpressing REV-ERBα in Huh-7 cells reduced SCD expression in a dose-dependent manner (Fig. 5e), consistent with our earlier data showing that endogenous REV-ERBα levels are low in Huh-7 cells (Fig. 3c). Cumulatively, these data show for the first time a role for REV-ERBα to regulate SCD in human hepatocytes.

As SCD is rate limiting for HCV infection^31,32^, we investigated the contribution of this pathway to REV-ERB regulation of HCV replication. Oleic acid is the final product of SCD and can restore de novo lipogenesis pathways in cells lacking SCD. Supplementing the culture media with oleic acid had a negligible effect on HCV replication (Supplementary Figure 7) but reduced the anti-viral activity of SR9009 and control SCD inhibitor (Fig. 5f). Furthermore, CRISPR knock-down (KD) of SCD in HCV replicon cells inhibits viral replication and reduced the anti-viral activity of SR9009 and SCDi (Fig. 5g), demonstrating that REV-ERB perturbation of SCD expression contributes to its anti-viral activity.

**Fig. 6:**
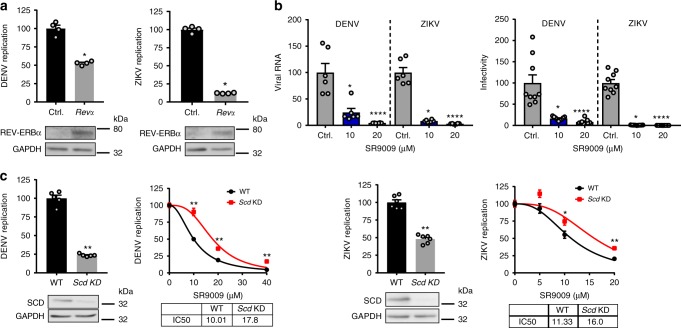
REV-ERB inhibits DENV and ZIKV replication. **a** Overexpressing REV-ERBα inhibits DENV and ZIKV replication. Huh-7 cells harbouring sub-genomic DENV RNA or ZIKV-Nanoluc were transfected with REV-ERBα expression plasmid or Ctrl empty vector (2 µg). DENV or ZIKV replication was assessed by measuring luciferase activity 48 h later and data expressed relative to untreated Ctrl cells (mean ± S.E.M., *n* = 4, Mann–Whitney statistical test). REV-ERB expression was confirmed by western blotting. **b** REV-ERB agonist SR9009 inhibits DENV and ZIKV infection. Huh-7 cells were infected with DENV2 16681 at a MOI of 0.1 and treated with SR9009 for 48 h (*n* = 3). Extracellular viral RNA and the infectious titre of secreted virus was quantified and the data expressed relative to untreated Ctrl cells, where the mean titre of virus secreted from untreated cells was 1.6 × 10^5^ pfu/ml. Huh-7 cells were infected with ZIKV MP1751 at an MOI of 0.1 and treated with SR9009 for 48 h. Extracellular RNA and infectious titre was quantified and the data expressed relative to untreated cells, where the mean titre of virus secreted from untreated cells was 2.9 × 10^6^ pfu/ml. **c** A role for SCD in REV-ERB agonist inhibition of DENV and ZIKV replication. DENV replicon cells or ZIKV-Nanoluc infected cells were transfected with CRISPRs targeting exons 2 and 3 of SCD or a scrambled guide RNA and 24 h later treated with SR9009. SCD expression was measured by western blotting and the dose of REV-ERB agonist required to inhibit DENV or ZIKV replication by 50% (IC_50_) in WT or KD cells determined (mean ± S.E.M., *n* = 6, Mann–Whitney statistical test)

As the replication of many flaviviruses is dependent on fatty acid biosynthesis^33^, we investigated a role for REV-ERB in the replication of DENV and ZIKV. Transient expression of REV-ERBα in Huh-7 cells using a dose of plasmid previously shown to inhibit HCV replication significantly reduced DENV and ZIKV replication (Fig. 6a). Treating Huh-7 cells with REV-ERB agonist SR9009 reduced DENV or ZIKV infection, as assessed by measuring secreted levels of viral RNA and infectious particles (Fig. 6b). Furthermore, CRISPR KD of SCD in DENV or ZIKV-infected Huh-7 cells reduced viral replication and the anti-viral activity of SR9009 (Fig. 6c), highlighting SCD as a common pathway for REV-ERB to regulate this family of viruses.

## Discussion

**Fig. 7:**
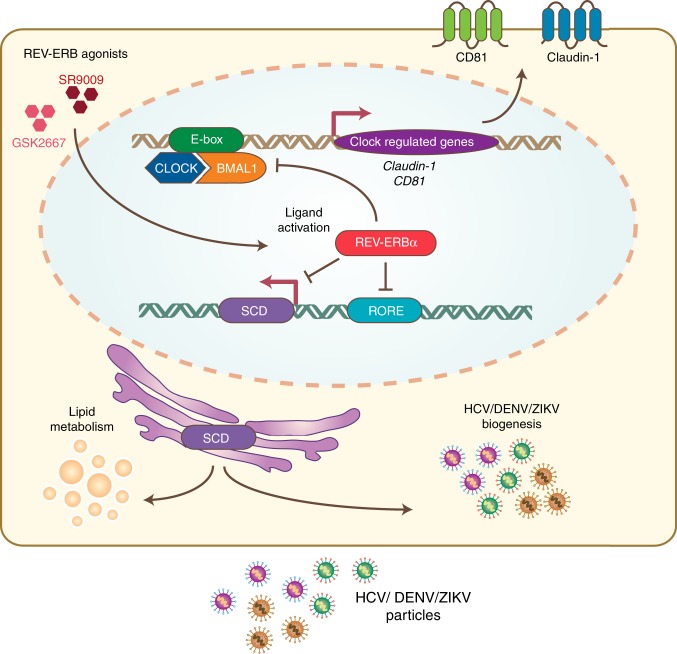
Model of circadian clock components regulating HCV, DENV and ZIKV replication. The circadian activator BMAL1 regulates HCV entry into hepatocytes through modulating viral receptors CD81 and claudin-1 expression. Activating REV-ERB with synthetic agonists or protein overexpression inhibits HCV, DENV or ZIKV RNA replication via modulating SCD and subsequent release of infectious particles

In this study, we examined the interplay between the circadian regulatory pathways and the replicative life cycle of HCV, DENV and ZIKV. Our results provide the first mechanistic evidence for circadian gating of HCV entry into hepatocytes via the regulation of entry receptors CD81 and claudin-1, providing a potential mechanism for our earlier observation reporting a time-of-day dependence in HCV re-infection kinetics following liver transplantation^34^. We discovered a new role for REV-ERB to regulate HCV, DENV and ZIKV RNA replication by repressing SCD expression (Fig. 7). Given the wide number of target genes that REV-ERB can regulate, we cannot exclude the possibility of additional pathways contributing to its anti-viral activity. However, our data provide the first evidence that pharmacological modulation of circadian pathways can inhibit viral replication. Targeting host pathways that are essential to virus replication can provide therapies that generate limited resistance and such chronotherapies may work synergistically with DAAs.

Our data showing rhythmic expression of CD81, claudin-1 and occludin transcripts in Huh-7 cells along with reduced CD81 and claudin-1 protein expression and HCVpp entry into *Bmal1* KO cells, support an interplay between BMAL1 and REV-ERB to regulate HCV entry into hepatocytes. Analysis of published ChIP-seq datasets^35,36^ from murine liver failed to identify BMAL1 binding to CD81 or claudin-1 promoter regions. However, claudin-1 and occludin mRNAs show a rhythmic expression in murine liver^37^ and claudin-1 was reported to be among the top upregulated genes in *Rev-erba* KO mice^18^. Du et al. reported that CD81 transcription was rhythmic in murine liver in phase consistent with REV-ERB target, but this did not result in rhythmic mRNA accumulation possibly due to high stability of the transcript^38^. Recent reports demonstrating a role for CD81 in the assembly and budding of human immunodeficiency virus and influenza A viruses^39,40^ highlight the wider implications of our observation that CD81 is circadian regulated for other human pathogens.

Our observation that inhibiting miR-122 reduced the anti-viral activity of REV-ERB agonists for HCV suggests that circadian regulation of HCV replication is in part miR-122 dependent. Of note, Esau and colleagues reported that miR-122 regulates cholesterol and lipid metabolism where silencing miR-122 in mice reduced the mRNA levels of key genes that regulate lipid metabolism, including SCD and fatty acid synthase (FASN)^28^. Our observation that a REV-ERB agonists inhibits HCV replication independent of miR-122 binding to the viral RNA, suggests an indirect modulation of HCV replication via perturbing lipid metabolism.

Recent studies reporting increased replication of herpes, influenza, respiratory syncytial virus and parainfluenza type 3 viruses in *Bmal1*-/- mice suggest that BMAL1 negatively regulates viral infection^8,9^. However, these studies did not address the underlying mechanism. Our observation that HCV, DENV and ZIKV show reduced replication in *Bmal1* KO hepatocytes highlights the diverse roles of BMAL1 in the life cycle of different virus families that requires further investigation. In vivo small animal models for studying HCV, DENV or ZIKV replication frequently use immunocompromised mice^41–43^ that limit studies to evaluate the immunomodulatory role of BMAL1 or REV-ERB in the viral life cycle.

Since virus infection, replication and particle assembly are dependent on cellular membranes, we examined the lipid composition of SR9009-treated Huh-7 cells and observed changes in both neutral (particularly triglycerides) and phospholipids. Notably, the lipidomic analysis highlighted selective changes in lipid molecular species, rather than a generalised change suggesting selective effects on lipid turnover. Of particular note was the 60% reduction in total cellular phosphatidic acid concentration, reflected almost exclusively in the mono- and di-unsaturated species (Supplementary Figure 8). This reduction is an expected consequence of SCD downregulation reducing monounsaturated fatty acid generation and is likely to induce a profound effect on cellular membranes. Phosphatidic acid is unique in that its small, highly charged head group is close to its glycerol backbone, allowing it to induce a high membrane curvature, which may play a role in viral RNA replication and particle assembly. In contrast, the unsaturated phosphatidic acids are more loosely packed in the membrane and induce less curvature. Consequently, we propose that the selective loss of mono- and di-unsaturated phosphatidic acid species will have a major effect on membrane structure and intracellular viral function.

Ultrastructural and functional studies show a role for SCD in the genesis of viral replication compartments or ‘replication factories’ in HCV, DENV and ZIKV^44^ but also West Nile^33^, human immunodeficiency virus^45^, respiratory syncytial virus and other respiratory viruses^46^, extending the significance of our observations to a wider spectrum of human pathogens. These viral replication factories serve multiple purposes: (1) spatial separation of different steps in the viral replication cycle, namely, RNA translation, replication and packaging of viral genomes into particles to prevent interference; (2) enabling a high local concentration of viral replicase complex components and metabolites such as nucleotides to maximise RNA amplification and (3) protecting newly synthesised viral RNA from innate immune surveillance. To date, our understanding of the role circadian factors play in viral replication at a cellular level is limited. Our findings show new pathways for the circadian network to impact multiple stages of the HCV, DENV and ZIKV replication cycles. As the transcriptional clock mechanism is universal and exists in all cells of the body, our study has implications for the circadian regulation of many viruses that rely on host metabolic activities to replicate^33,45–47^.

## Methods

### Cell culture

The human hepatoma cell line Huh-7 (gift from C. Rice, Rockefeller University, NY) and HCV sub-genomic replicon line A2-Luc^48^ (gift from R. Thimme, University of Freiburg, Germany) were maintained in Dulbecco’s modified Eagle’s medium (DMEM)/10% foetal bovine serum (FBS)/1% nonessential amino acids/1% penicillin/streptomycin (Invitrogen, Carlsbad, CA). Huh-7 cells were synchronised by treating with culture medium containing 50% FBS for 1 h. *Bmal1* KO Huh-7 clones were generated by transfecting a pool of three BMAL1 CRISPR/Cas9 KO plasmids (Santa Cruz Biotechnology, UK) followed by fluorescence activated cell sorting (FACs) and clonal expansion. LLC-MK2 and AFRIMS kidney cells (ATCC, UK) were maintained in M199 medium/10% FBS/1% penicillin/streptomycin. Vero epithelial kidney cells (ATCC, UK) were maintained in DMEM/10% FBS/1% penicillin/streptomycin.

### Reagents and antibodies

Following reagents were purchased from commercial suppliers: REV-ERBα expression plasmid (Origene, UK); SCD promoter-luc plasmid (Genecopoeia, UK); REV-ERB agonist SR9009 (Calbiochem, US); Daclatasvir and Sofosbuvir (Selleckchem, US) and SCD inhibitor A939572 (BioVision, US). REV-ERB agonist GSK2667 was synthesised at University of Birmingham^10^. All drugs were dissolved in dimethyl sulfoxide (DMSO) and their cytotoxicity determined by a Lactate dehydrogenase (LDH) assay (Promega, UK). The following primary antibodies were used: anti-NS5A 9E10 (1 µg/mL, C. Rice, Rockefeller University), anti-CD81 (1 µg/mL, 2.131)^49^, anti-claudin-1 (1 µg/mL, Abcam, UK), anti-occludin (2 µg/mL, Invitrogen, UK), anti-BMAL1 (1 µg/mL, Abcam, UK), anti-REV-ERBα (1 µg/mL, Thermo Fisher Scientific, UK); anti-SCD (1 µg/mL, Abcam, UK) and anti-GAPDH (1 µg/mL, Cell Signaling, US). Uncropped original western blots are shown in Supplementary Figure 9. Fluorescent Alexa Fluor 488-conjugated anti-mouse secondary antibodies were obtained from Invitrogen, UK.

The BMAL1 promoter was amplified from genomic DNA using forward primer: 5ʹ-CCGCTCGAGGGGACAACGGCGAGCTCGCAG-3ʹ and reverse primer: 5ʹ-CCCAAGCTTCGGCGGCGGCGGCGGCAAGTC-3ʹ and cloned into the pGL3 luciferase reporter vector (Promega, UK). Lenti-shRev-Erbα construct was a gift from Dr. B. Grimaldi, University of Genoa, Italy. To generate SCD KD cells, CRISPR guide RNA (gRNA) forward and reverse sequences were designed using CRISPR Finder (https://www.sanger.ac.uk/htgt/wge/find_crisprs) with overhang sequences containing a *Bbs*I restriction site^50^. The gRNA sequences were annealed and cloned into *Bbs*I-digested pSpCas9(BB)-2A-Puro (PX459) V2.0 DNA (Addgene plasmid #62988; deposited by Dr. Feng Zhang). The CRISPR gRNA plasmid products were sequenced to confirm successful cloning. SCD exon 2 gRNA sequences: exon 2 forward—5ʹ-CACCGGCCTTCCTTATCCTTGTAGG-3ʹ and exon 2 reverse 5ʹ-AAACCCTACAAGGATAAGGAAGGCC-3ʹ. SCD exon 3 gRNA sequences: exon 3 forward—5ʹ-CACCGGCAGCCGAGCTTTGTAAGAG-3ʹ and exon 3 reverse- 5ʹ-AAACCTCTTACAAAGCTCGGCTGCC-3ʹ. All primers used are detailed in Supplementary Table 1.

### HCVcc/HCVpp genesis and infection

Plasmids encoding HCV SA13/JFH and J6/JFH were transcribed to generate viral RNA that was electroporated into Huh-7 cells^51^. Infected cells were fixed with ice-cold methanol, stained for viral antigen expression with anti-NS5A (9E10) and an isotype-matched Alexa-488 conjugated IgG. Viral antigen-expressing cells were enumerated using a fluorescent microscope. HCV RNA levels were assessed by quantitative reverse transcription polymerase chain reaction (qRT-PCR). Luciferase reporter pp expressing HCV envelope glycoproteins (HCVpp), or no-glycoprotein controls, were generated in 293T cells^20^ using a plasmid encoding a HIV provirus expressing luciferase and viral envelope glycoproteins from HCV lab strains H77 and from patient-derived clones^52^. Circadian synchronised or 24 h drug-treated Huh-7 cells were inoculated with pp for 1 h, lysed 24 h later and luciferase activity measured.

### DENV and ZIKV infection

DENV2 strain 16681 was grown in 6/36 cells, concentrated by precipitation with 10% weight per unit volume (w/v) poly(ethyleneglycol) (PEG) MW 8000 (Sigma, UK)/0.6% sodium chloride. Huh-7.5 cells were seeded into 24-well plates and 24 h later infected with DENV2 16681 at a multiplicity of infection (MOI) of 1 for 90 min at room temperature (RT). Unbound virus was removed by washing and cultures treated with SR9009 or control DMSO and incubated for 48 h. Supernatants were harvested and the infectious titre determined by plaque assay on LLC-MK2 cells. Viral RNA was extracted from the supernatant using the Qiagen Viral RNA mini kit according to the manufacturer’s protocol.

ZIKV strain MP1751 (PHE, UK) was propagated in Vero cells, concentrated by precipitation with 8% w/v PEG in NTE buffer. Huh-7.5 cells were infected with ZIKV strain MP1751 at an MOI of 0.1 for 90 min at RT, unbound virus was removed by washing and the cultures treated with SR9009 or control DMSO and incubated for 48 h. Supernatants were harvested and the infectious titre determined by plaque assay on Vero cells. Viral RNA was extracted from the supernatant using the Qiagen Viral RNA mini kit according to the manufacturer’s protocol.

The pCCl-SP6- ZIKV-Nanoluc plasmid^53^ was used as a template to generate genomic DNA by PCR using the following primers: forward 5ʹ-CGATTAAGTTGGGTAACGCCAGGGT-3ʹ and reverse 5ʹ-TAGACCCATGGATTTCCCCACACC-3ʹ. The DNA was transfected into Vero E6 cells (ATCC, CCL-81) using Lipofectamine 2000 as per the manufacturer’s instructions (Thermo Fisher Scientific). Virus stocks were titrated in the A549/BVDV-Npro cell line^54^. Huh-7.5 cells were infected with ZIKV-Nanoluc at an MOI of 0.1 and treated with SR9009 or DMSO vehicle alone for 48 h and Nluc activity measured using the Nano-Glo kit (Promega).

### Real-time reverse transcription PCR

Purified total RNA samples were tested for HCV RNA expression (Primer Design Ltd, UK) in a qRT-PCR as per the manufacturer’s guidelines (CellsDirect Kit; Invitrogen, UK) in an MxPro-3000 PCR machine (Aligent, US). Comparison of a panel of 12 housekeeping genes (GeNorm kit, Primer Design Ltd. UK) identified GAPDH as a stably expressed reference gene. Primer sets for BMAL1, REV-ERBα, CD81, claudin-1, occludin and common housekeeping genes were purchased from Thermo Fisher Scientific, UK. For SCD a primer set consisting of forward: 5ʹ-CTCTGCTACACTTGGGAGCC-3ʹ and reverse: 5ʹ-GAGCTCCTGCTGTTATGCCC-3ʹ was used in a SYBR green qRT-PCR (Qiagen, UK).

DENV and ZIKV RNA was assayed by qRT-PCR on an Applied Biosystems 7500 real-time PCR system using the Verso 1-step RT-PCR kit with Thermo-start Taq (Applied Biosystems) as per the manufacturer’s guidelines. The primer and probe sequences were adapted from previously published methods^55,56^. For DENV, the NS5 primer set consisted of forward 5ʹ-ACAAGTCGAACAACCTGGTCC-3ʹ, reverse 5ʹ-GCCGCACCATTGGTCTTCTC-3ʹ and probe 5ʹ-(6FAM) CCAGTGGAATCATGGGAGGAAATCCCA(TAM)-3ʹ. For ZIKV, the envelope primer set consisted of forward 5ʹ-TCGTTGCCCAACACAAG-3ʹ, reverse 5ʹ-CCACTAATGTTCTTTTGCAGACAT-3ʹ and probe 5ʹ-(6FAM) AGCCTACCTTGACAAGCAATCAGACACTCAA(TAM)-3ʹ. Secreted DV RNA was quantified using a standard curve generated from high titre viral RNA isolated from C6/36 grown DENV2. Secreted ZIKV RNA was quantified using a standard curve generated from an RNA oligonucleotide comprising the 77 bases of ZIKV RNA targeted by the assay primers.

### HCV and DENV sub-genomic replicons

Plasmids encoding the HCV and DENV sub-genomic replicons were generated^57,58^. The HCV L-GDD con1 (genotype 1b), JFH-1-luc (genotype 2a) and S52-ΔN (genotype 3a) were linearised with *Xba*I (New England Biolabs, UK), treated with Mung Bean nuclease (NEB, UK) and the linearised templates used to transcribe RNAs^24^. Plasmid pDVRepPAC-LUC encodes a firefly luciferase (LUC) gene in place of DENV2 C, prM and E genes and a NS2B-NS3 cleavage site at the C-terminus enables cleavage from the rest of the polyprotein. In all, 2 μg of HCV or DENV RNA was electroporated into 4 × 10^6^ cells and allowed to recover for 48 h before treating with REV-ERB ligands. Huh-7 cells stably expressing HCV RNA were generated from N17 plasmid^59^. Cells were seeded in 96-well plates, incubated with Vivo-Glo (Promega, UK) and monitored in real-time at 30-min intervals over a 24-h period on a microplate reader (Clariostar, BMG Labtech, UK).

### Microarray and ChIP-seq analyses

Microarray datasets for SR9009 treated Huh-7 cells (20 µM, 24 h treatment, *n* = 3) were obtained from Oxford Gene Technology, UK. Differentially expressed genes were filtered using the following parameters: *p*-value < 0.05, log_2_FC > 0.5 or log_2_FC < –0.5. KEGG profiling^60^ was used to perform pathway mapping and determine significantly enriched pathways using hypergeometric tests. Metabolic-related pathways were extracted and represented as bar charts with the colour of each bar indicative of its *p*-value. ChIP-seq datasets for murine REV-ERBα and REV-ERBβ were obtained from Gene Expression Omnibus where peak detection was performed using the HOMER tool. BedGraphs are visualised using the Integrative Genomics Viewer (IGV) tool^61^ overlaying the mouse genome (mm10).

### Analysis of cellular triacylglycerol fatty acids and phospholipids

Total lipids from mock or SR9009 (20 µM)-treated Huh-7 cells were extracted using the Folch method^62^. The triacylglycerol fraction was separated using solid phase extraction columns and fatty acid methyl esters (FAMEs) prepared by incubating with methanolic sulphuric acid at 80 °C for 2 h^63^. Gas chromatography was performed using an Agilent 6890N GC (Agilent Technologies, Stockport, UK)^64^ and individual fatty acid peaks identified by reference to known FAMEs and fatty acid peak areas converted to mol (%). Phospholipids were analysed by mass spectrometry^65^ and lipids resuspended in chloroform/methanol (1:1) prior to injection into a Shimadzu Prominence 20 AD system (Shimadzu, Kyoto, Japan). Chromatographic separation was achieved upon a Cogent HPLC column (150 × 2.1 mm, 4 µm particle size) column kept a 40 °C. Mobile phase A: isopropanol/hexane/100 mM ammonium formate (58:40:2), mobile phase B: isopropanol/hexane/100 mM ammonium formate with 0.5% of formic acid (50:40:10). The gradient started at 10% B (5 min), with an increase to 90% B for 5 min, 100% B was reached after an additional 10 min and held for 7 min before re-equilibrating with 10% B for 5 min. The flow rate was maintained constant at 200 µL/min. Accurate mass (with an error below 5 ppm) was acquired on an Orbitrap Elite mass spectrometer (Thermo Fisher Scientific, USA). Source parameters for negative polarity were: capillary temperature 325 °C; source heater temperature 325 °C; sheath gas 10 AU; aux gas 5 AU; sweep gas 5 AU. Source voltage was 3.2 kV. Full scan spectra in the range of m/z 200–1000 were acquired at a target resolution of 240,000 (FWHM at m/z 400). Data were analysed using Lipid Data Analyzer (2.6.0–2) software.

### Proteomic analysis

Three biological replicates of control and SR9009-treated Huh-7 cells were lysed in RIPA buffer and proteins separated using NuPAGE 4–12% bis-tris gels (Invitrogen). Peptides obtained from selected proteins (CD81, claudin-1, occludin, SR-BI, occluding, SCD) using in-gel trypsin digestion (tryptic digestion kit, Thermo) were detected and quantified using Liquid chromatography tandem mass spectrometry (LC-MS/MS) methods. Peptides were desalted using an online C18 trap column (PepMap 100, 5 µm particle size, 300 µm i.d. × 5 mm, Thermo Fisher Scientific) at a flow rate of 10 µL/min for 4 min. The nano LC system Dionex Ultimate 3000 (RSLC nano system) and a nano analytical C18 reversed phase column (PepMap), dimensions 75 µm × 50 cm, 2 µm particle size (Thermo Fisher Scientific) were used, with a flow rate of 250 nL/min at 45 ^o^C ( ± 2 ^o^C). The mobile phase was solvent A: 0.1% v/v formic acid in water, and solvent B: 0.1% v/v formic acid in acetonitrile/water (80:20 v/v). A four step-gradient (0–4.5 min: 2% B, 4.5–105 min: 25% B, 105–125 min: 40% B and 125–131 min: 95% B) was used to separate peptides on the nano analytical column. Mass spectrometry was performed using a bench top Q Exactive hybrid quadrupole-Orbitrap mass spectrometer (Thermo Scientific) for data-dependent acquisition (DDA) and parallel reaction monitoring (PRM). DDA was used for detection and library creation of peptides for the purpose of PRM-based quantitation. DDA method settings: Chromatographic peak width: 12 s, the full MS conditions used—resolution: 70,000, AGC target: 3e6, maximum injection time (IT): 60 ms, scan range: 375–1500 m/z. The dd-MS^2^ conditions—resolution: 17,500. The AGC target conditions—1e^5^, maximum IT: 60 ms, loop count: 10 (i.e., Top 10), isolation width: 2.0 m/z, fixed first mass: 100.0 m/z, and the data-dependent (dd) settings—minimum AGC target: 2e^3^, intensity threshold: 3e^4^, charge exclusion: unassigned, 1, > 8, peptide match; preferred, dynamic exclusion: 20 s. Normalised collision energy (NCE) of 27 was used for fragmentation of peptides in a high-energy collision dissociation (HCD) cell. PRM method settings: global settings—user role: advanced; lock mass: best; chromatographic peak width: 20 s; full MS-SIM setting—resolution: 17,500; AGC target: 5e;^4^ maximum IT: 20 ms; t-MS^2^ settings—polarity: positive; in-source collision induced dissociation (CID): 0.0 eV; default charge: 2; inclusion: on; microscan: 1; resolution: 70,000; AGC target: 2e;^5^ maximum IT: 200 ms; MSX count: 1; isolation window: 1.6 m/z; normalised collision energy (NCE): 27; spectrum type: profile. The MS tune file for nano flow rate at 250 nL/min was used with the following settings: scan type: full MS-SIM, scan range: 350–2000 m/z, fragmentation: none, resolution: 70,000, polarity: positive, microscan: 1, AGC target: 1e^6^, maximum IT: 100, sheath gas flow: 0, aux gas flow: 0, sweep gas flow: 0, spray voltage: 2.3 kV, capillary temperature: 320 ^o^C, S-lens RF level: 50. Data acquired using DDA were processed using the Mascot search engine against human Uniprot protein database. Mascot search results were uploaded to Skyline (version 4.1.0.18169, 64-bit, Seattle, USA) to create a reference library. PRM data were processed using Skyline to quantify peptides in different samples.

### Statistical analysis

Statistical analyses were performed in Graph Pad Prism 7 (GraphPad, USA) using Mann–Whitney’s test or Kruskal–Wallis one-way analysis of variance (ANOVA) with Dunn’s test (for multiple comparisons), except where stated otherwise, with a *p*-value of < 0.05 considered statistically significant (*p ≤ 0.05, **p ≤ 0.01, ***p ≤ 0.001, ****p ≤ 0.0001).

### Reporting summary

Further information on experimental design is available in the Nature Research Reporting Summary linked to this article.

## Supplementary information

Supplementary Information

Description of Additional Supplementary Files

Supplementary Data 1

Reporting Summary

## Data Availability

The metabolomics data have been deposited to the EMBL-EBI MetaboLights database (DOI: 10.1093/nar/gks1004. PubMed PMID: 23109552) with the identifier MTBLS792. The mass spectrometry proteomics data have been deposited to the ProteomeXchange Consortium via the PRIDE [1] partner repository with the dataset identifier PXD011721 and 10.6019/PXD011721, and can be accessed via: ftp://PASS01292:YF2765va@ftp.peptideatlas.org/. The microarray data from SR9009 treated Huh-7 cells have been deposited at NCBI GEO with the identifier GSE123748 and can be accessed via http://www.ncbi.nlm.nih.gov/geo/query/acc.cgi?acc=GSE123748. The authors declare that all other data supporting the findings of this study are available within the article and its Supplementary Information files, or are available from the authors upon request.
