# Supplementary material for: The circadian clock components BMAL1 and REV-ERBα regulate flavivirus replication
Source: Nat Commun. 2019 Jan 22;10:377. doi: 10.1038/s41467-019-08299-7 (PMC6343007; doi:10.1038/s41467-019-08299-7)
Supplement: Supplementary file 1 — Supplementary Information [file 41467_2019_8299_MOESM1_ESM.pdf]

Supplementary figure 1

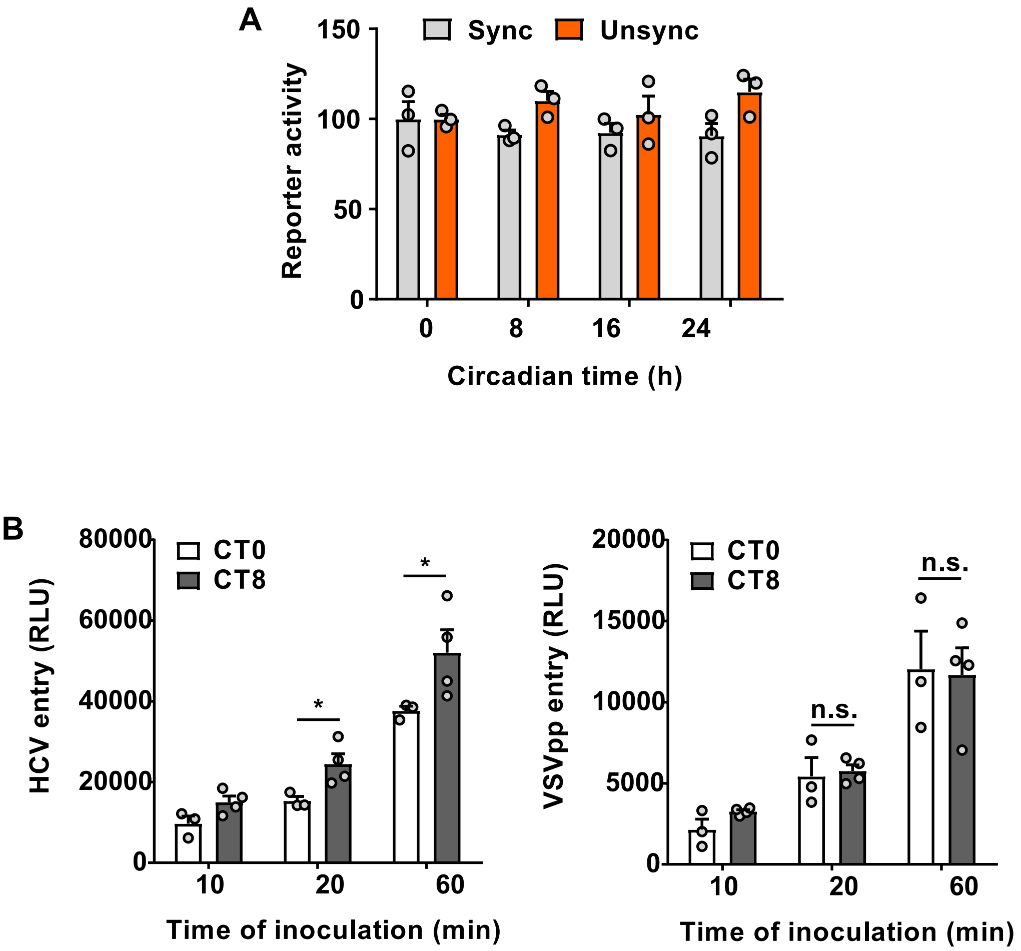

A. Serum shock does not affect reporter activity. Huh-7 cells were transfected with lentiviral reporter NL4.3R-Env- Luc as detailed in materials and methods and 24h later treated with 50% FBS containing media (sync) or normal media (unsyn) for 1h. Luciferase activity was measured at 8h intervals for 24h. **B. Increased HCV entry at CT8 compared with CT0.** Huh-7 cells were synchronized and at CT0 or CT8 inoculated with HCVpp or VSVpp for 10, 20 or 60 min and luciferase activity measured 24h later (mean ± S.E.M., n = 3 or 4, Mann–Whitney statistical test).

Supplementary figure 2

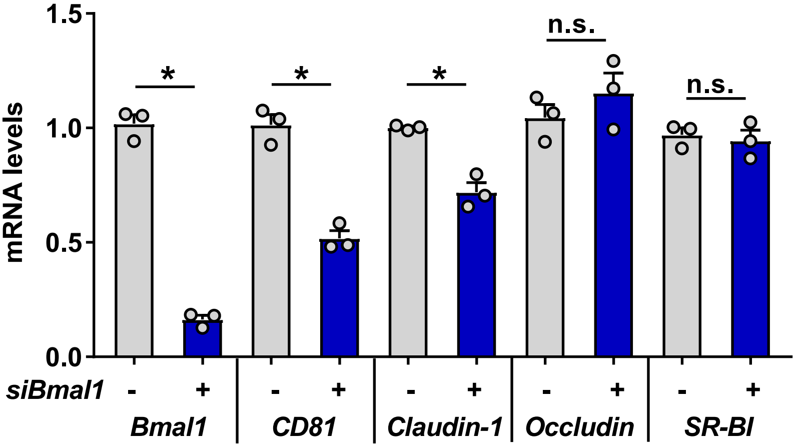

Silencing Bmal1 reduces CD81 and Claudin-1 mRNA levels. Real-time qPCR was performed to measure the mRNA levels of HCV entry receptors and Bmal1 in siRNA silenced cells 48h post transfection. Data is expressed relative to an irrelevant siRNA control (mean ± S.E.M., n = 3, Mann–Whitney statistical test).

Supplementary figure 3

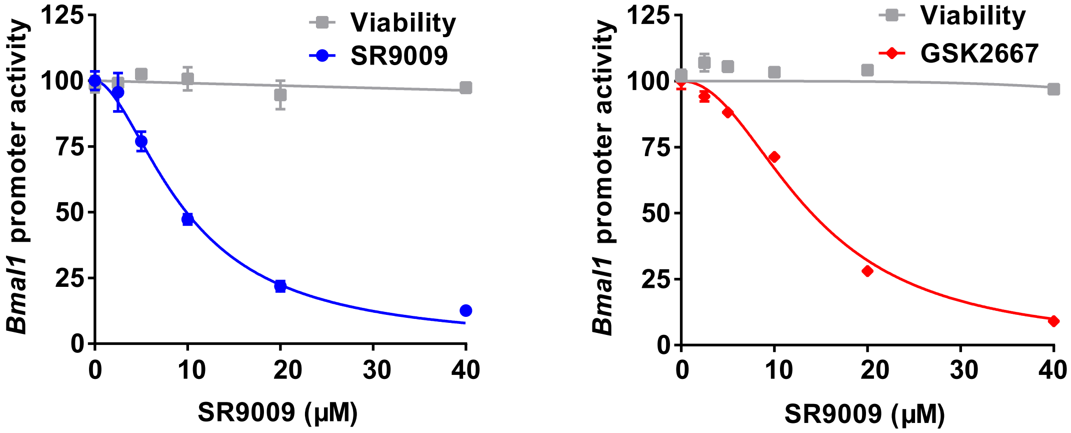

REV-ERB agonists inhibit Bmal1 promoter activity. Huh-7 cells stably expressing Bmal1-luciferase promoter were treated with REV-ERB ligands SR9009 or GSK2667 for 24h and luciferase activity measured. Cytotoxicity was determined using an LDH assay (mean ± S.E.M., n = 3).

Supplementary figure 4

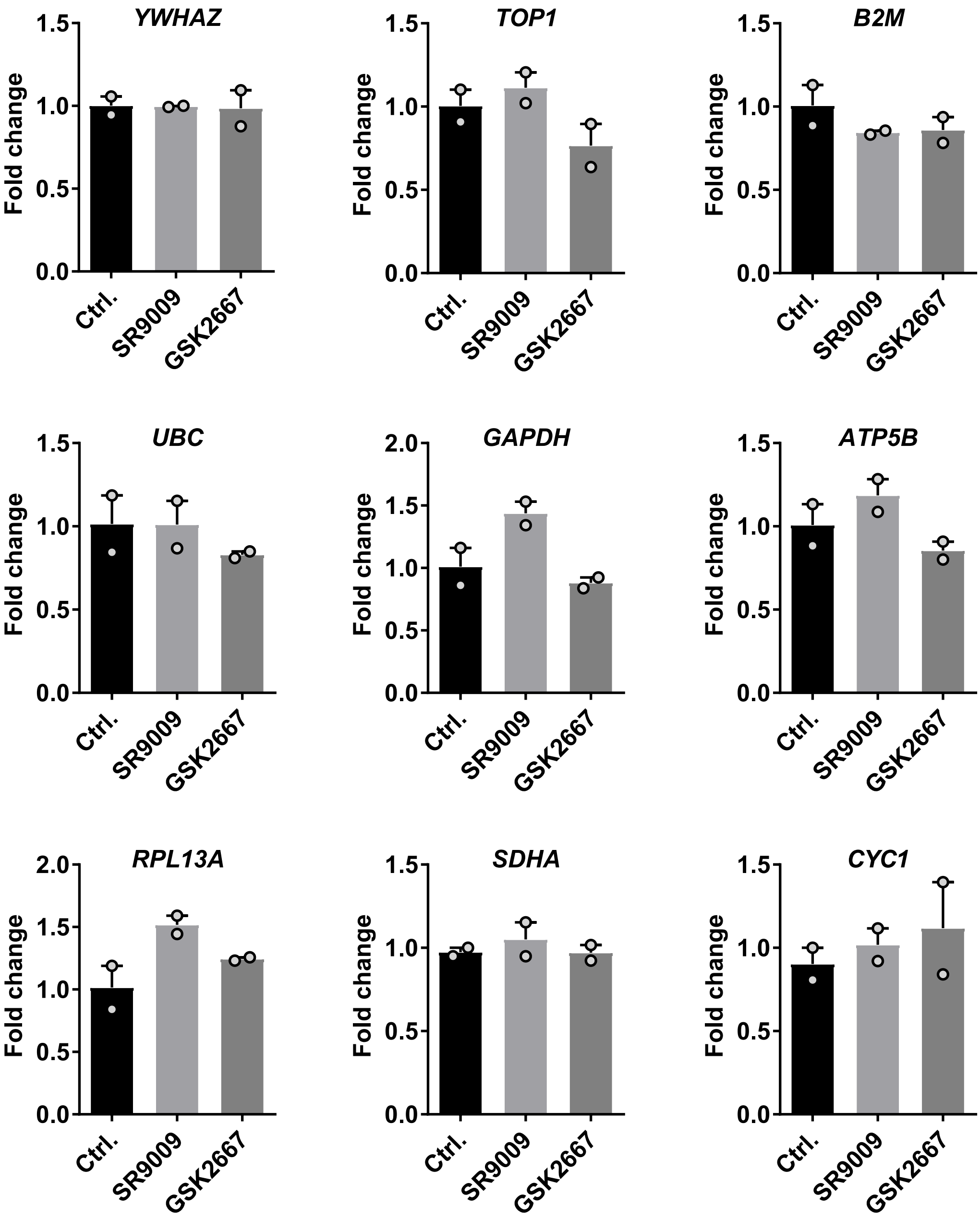

Transcription profiling of housekeeping genes in REV-ERB agonist treated cells. Huh-7 cells were treated with Rev-Erb agonists (20 µM) for 24h following qPCR quantification of nine common housekeeping genes. Data is expressed relative to untreated Ctrl cells, mean ± S.E.M., n = 2, Kruskal- Wallis One-Way ANOVA with Dunn's test.

Supplementary figure 5

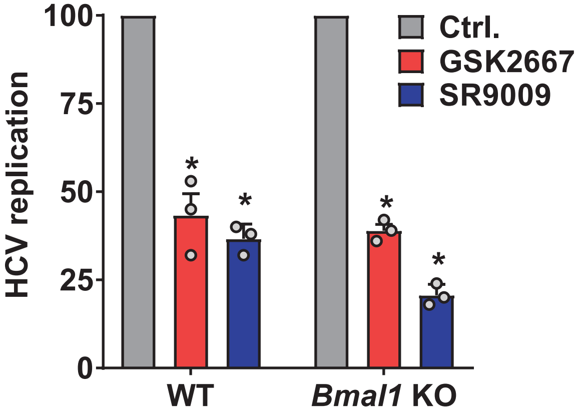

Anti-viral activity of REV-ERB agonists is independent of BMAL1. Parental (WT) or Bmal1 KO Huh-7 cells were electroporated with sub- genomic HCV JFH-1 RNA and 24h later treated with REV-ERB agonists for 24h. HCV replication was assessed by measuring luciferase activity and the data expressed relative to Ctrl untreated cells (mean ± S.E.M., n = 3, Kruskal- Wallis ANOVA with Dunn’s test).

Supplementary figure 6

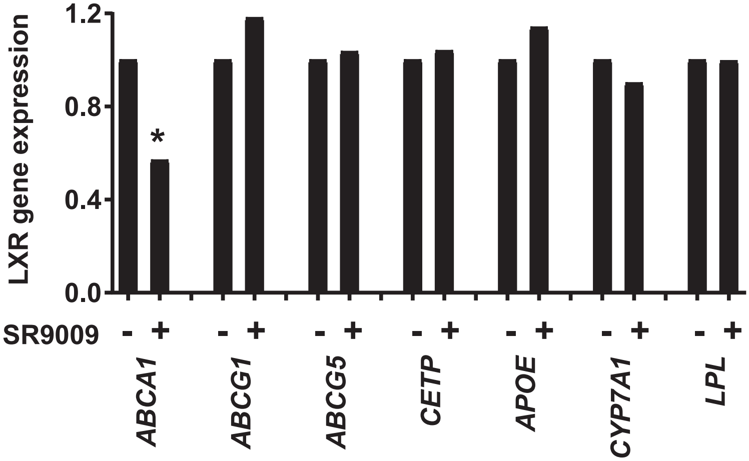

REV-ERB agonist SR9009 does not activate LXR target genes in Huh-7 cells. Relative expression of LXR target genes in Huh-7 cells following SR9009 (20 µM) treatment.

Supplementary figure 7

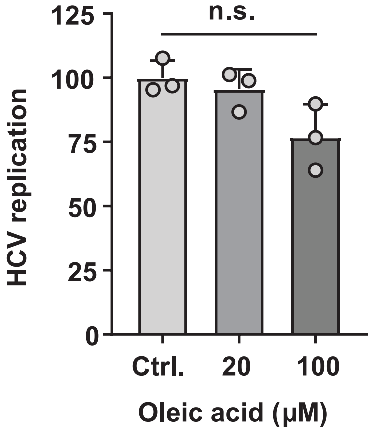

Oleic acid does not affect HCV replication. HCV replicon cells were treated with oleic acid at 20 or 100 µM for 24h and viral replication was determined by luciferase assay (mean ± S.E.M., n = 3, Mann–Whitney statistical test).

Supplementary figure 8

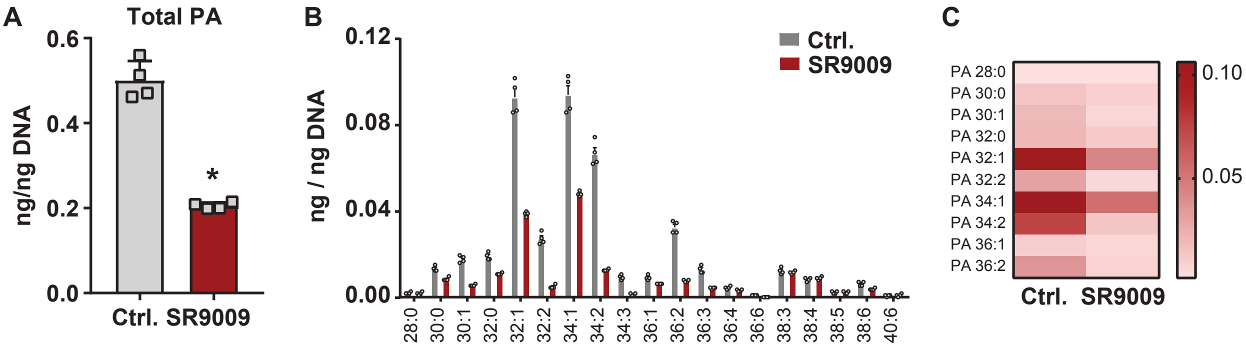

Phospholipid analysis of REV-ERB agonist SR9009 treated Huh-7 cells. A. Total cellular phosphatidic acid concentration (mean ± S.E.M., n = 4, Mann–Whitney statistical test).; B. PA Lipid species in control and SR9009 treated cells (mean ± S.E.M., n = 4); C. Mono- and di-saturated PA molecular species.

Supplementary figure 9

Figure 1F

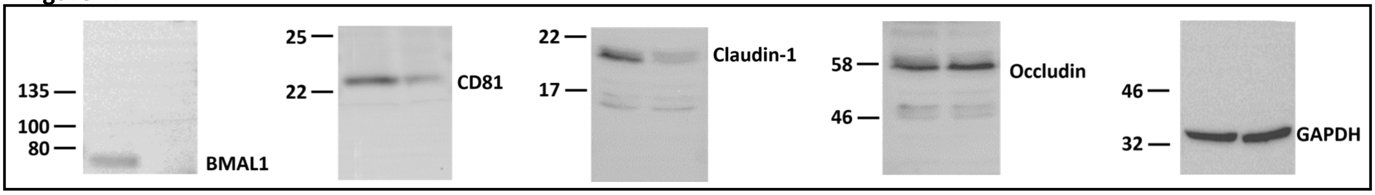

Figure 2B

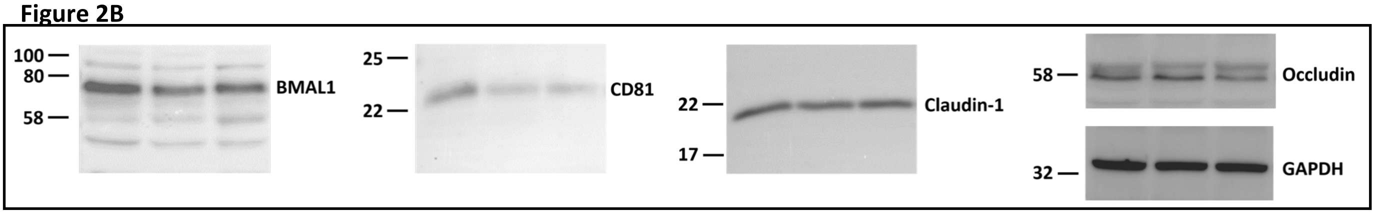

Figure 3A

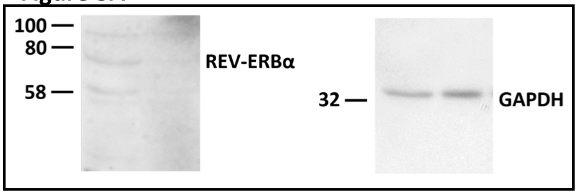

Figure 3C

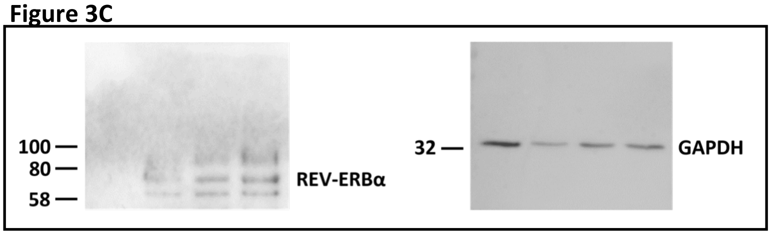

Figure 5E

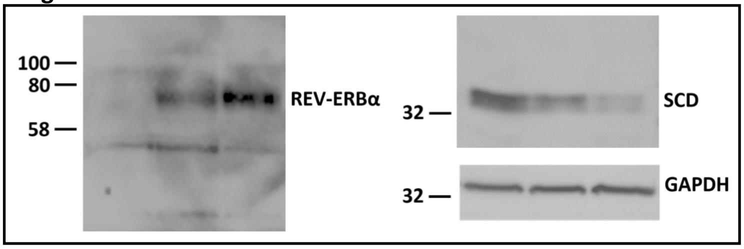

Figure 5G

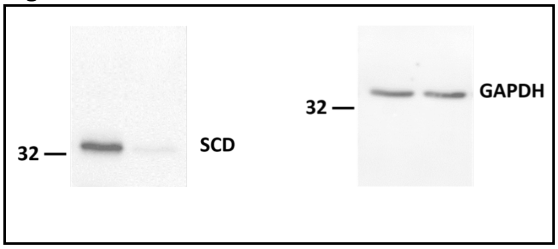

Figure 6A

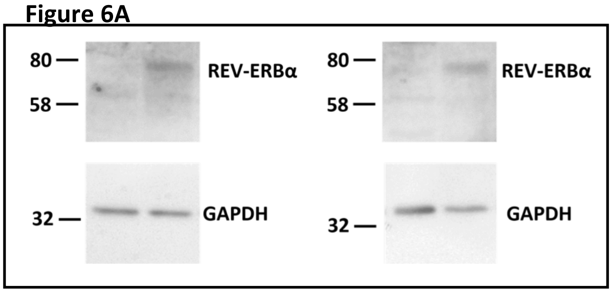

Figure 6C

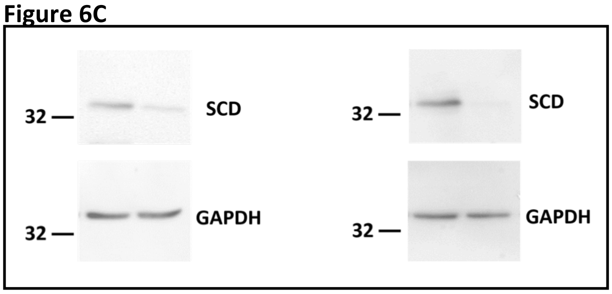

Uncropped scans of western blots

Supplementary table 1: Primers

Name	Forward (5' to 3')	Reverse (5' to 3')
SCD expression primer	CTCTGCTACACTTGGGAGCC	GAGCTCCTGCTGTTATGCCC
Expression primer sets for Bmal1, Rev-erba, CD81, claudin-1, occludin and common housekeeping genes were purchased from Thermo Fisher Scientific.		
Bmal1 promoter generation	CCGCTCGAGGGGACAACGGCGAGCTCGCAG	CCCAAGCTTCGGCGGCGGCGGCGGCAAGTC
SCD exon 2 gRNA sequences	CACCGGCCTTCCTTATCCTTGTAGG	AAACCCTACAAGGATAAGGAAGGCC
SCD exon 3 gRNA sequences	CACCGGCAGCCGAGCTTTGTAAGAG	AAACCTCTTACAAAGCTCGGCTGCC
pCCl-SP6-ZIKV-Nanoluc generation	CGATTAAGTTGGGTAACGCCAGGGT	TAGACCCATGGATTTCCCCACACC
DENV-NS5 primer	ACAAGTCGAACAACCTGGTCC	GCCGCACCATTGGTCTTCTC
	Probe 5' (6FAM) CCAGTGGAATCATGGGAGGAAATCCCA(TAM)-3'	
ZIKV-envelope primer	TCGTTGCCCAACACAAG	CCACTAATGTTCTTTTGCAGACAT
	Probe 5' (6FAM) AGCCTACCTTGACAAGCAATCAGACACTCAA(TAM)-3'.	
