# Supplementary material for: The circadian clock components BMAL1 and REV-ERBα regulate flavivirus replication
Source: Nat Commun. 2019 Jan 22;10:377. doi: 10.1038/s41467-019-08299-7 (PMC6343007; doi:10.1038/s41467-019-08299-7)
Supplement: Supplementary file 2 — Description of Additional Supplementary Files [file 41467_2019_8299_MOESM2_ESM.pdf]

File Name: Supplementary Data 1

Description: Top 100 genes regulated in SR9009 treated Huh-7 cells (20uM, 24h).
