# Supplementary material for: The circadian clock components BMAL1 and REV-ERBα regulate flavivirus replication
Source: Nat Commun. 2019 Jan 22;10:377. doi: 10.1038/s41467-019-08299-7 (PMC6343007; doi:10.1038/s41467-019-08299-7)
Supplement: Supplementary file 4 — Reporting Summary [file 41467_2019_8299_MOESM4_ESM.pdf]

nature research

Corresponding author(s): Jane McKeating

Reporting Summary

Nature Research wishes to improve the reproducibility of the work that we publish. This form provides structure for consistency and transparency in reporting. For further information on Nature Research policies, see Authors & Referees and the Editorial Policy Checklist.

Statistical parameters

When statistical analyses are reported, confirm that the following items are present in the relevant location (e.g. figure legend, table legend, main text, or Methods section).

n/a	Confirmed	
☐	☒	The exact sample size (n) for each experimental group/condition, given as a discrete number and unit of measurement
☐	☒	An indication of whether measurements were taken from distinct samples or whether the same sample was measured repeatedly
☐	☒	The statistical test(s) used AND whether they are one- or two-sided<br>*Only common tests should be described solely by name; describe more complex techniques in the Methods section.*
☒	☐	A description of all covariates tested
☒	☐	A description of any assumptions or corrections, such as tests of normality and adjustment for multiple comparisons
☐	☒	A full description of the statistics including central tendency (e.g. means) or other basic estimates (e.g. regression coefficient) AND variation (e.g. standard deviation) or associated estimates of uncertainty (e.g. confidence intervals)
☐	☒	For null hypothesis testing, the test statistic (e.g. F, t, r) with confidence intervals, effect sizes, degrees of freedom and P value noted<br>*Give P values as exact values whenever suitable.*
☒	☐	For Bayesian analysis, information on the choice of priors and Markov chain Monte Carlo settings
☒	☐	For hierarchical and complex designs, identification of the appropriate level for tests and full reporting of outcomes
☒	☐	Estimates of effect sizes (e.g. Cohen's d, Pearson's r), indicating how they were calculated
☐	☒	Clearly defined error bars<br>*State explicitly what error bars represent (e.g. SD, SE, CI)*

Our web collection on statistics for biologists may be useful.

Software and code

Policy information about availability of computer code

Data collection	Lipidomic data was analysed using Lipid Data Analyzer (2.6.0-2) software. Proteomic data were processed using the Mascot search engine against the human Uniprot protein database.
Data analysis	All statistical analyses were performed using GraphPad Prism 7

For manuscripts utilizing custom algorithms or software that are central to the research but not yet described in published literature, software must be made available to editors/reviewers upon request. We strongly encourage code deposition in a community repository (e.g. GitHub). See the Nature Research guidelines for submitting code & software for further information.

Data

Policy information about availability of data

All manuscripts must include a data availability statement. This statement should provide the following information, where applicable:

- Accession codes, unique identifiers, or web links for publicly available datasets
- A list of figures that have associated raw data
- A description of any restrictions on data availability

Data availability
The research materials generated and analysed in the current study are available via the following repositories : The metabolomics data have been deposited to the EMBL-EBI MetaboLights database (DOI: 10.1093/nar/gks1004. PubMed PMID: 23109552) with the identifier MTBLS792 : https://www.ebi.ac.uk/metabolights/MTBLS792. The mass spectrometry proteomics data have been deposited to the ProteomeXchange Consortium via the PRIDE [1] partner repository with the dataset identifier PXD011721 and 10.6019/PXD011721, and can be accessed via : ftp://PASS01292:YF2765va@ftp.peptideatlas.org/.

Field-specific reporting

Please select the best fit for your research. If you are not sure, read the appropriate sections before making your selection.

☒ Life sciences ☐ Behavioural & social sciences ☐ Ecological, evolutionary & environmental sciences

For a reference copy of the document with all sections, see nature.com/authors/policies/ReportingSummary-flat.pdf

Life sciences study design

All studies must disclose on these points even when the disclosure is negative.

Sample size	Sample size was chosen consistent with the previous literature utilising similar assays. For most experiments this was achieved using triplicate measurements. Where used, higher replicate numbers are indicated in the text.
Data exclusions	No data was excluded
Replication	Biological replicates were performed as described in the main text.
Randomization	No randomization was performed
Blinding	Blinding was not relevant to this study

Reporting for specific materials, systems and methods

Materials & experimental systems

n/a	Involved in the study	
☒	☐	Unique biological materials
☐	☒	Antibodies
☐	☒	Eukaryotic cell lines
☒	☐	Palaeontology
☒	☐	Animals and other organisms
☒	☐	Human research participants

Methods

n/a	Involved in the study	
☐	☒	ChIP-seq
☒	☐	Flow cytometry
☒	☐	MRI-based neuroimaging

Antibodies

Antibodies used	The following primary antibodies were used: anti-NS5A 9E10 (C. Rice, Rockefeller University), anti-CD81 (2.131)49, anti-claudin1 (Abcam, UK), anti-occludin (Invitrogen, UK), anti-BMAL1 (Abcam, UK), anti-REV-ERBα (Thermo Fisher Scientific, UK); anti-SCD (Abcam, UK) and anti-GAPDH (Cell Signaling, US). Fluorescent Alexa Fluor 488 conjugated anti-mouse secondary antibodies were obtained from Invitrogen, UK.
Validation	All commercially available antibodies were validated by their respective manufacturers

Eukaryotic cell lines

Policy information about cell lines

Cell line source(s)	All original cell lines were obtained from ATCC
Authentication	None of the cell lines were authenticated

Mycoplasma contamination | Mycoplasma testing was performed every month on all cell lines, and verified as negative.

Commonly misidentified lines (See ICLAC register) | None

ChIP-seq

Data deposition

☒ Confirm that both raw and final processed data have been deposited in a public database such as GEO.

☒ Confirm that you have deposited or provided access to graph files (e.g. BED files) for the called peaks.

Data access links
May remain private before publication.
TBA

Files in database submission
TBA

Genome browser session (e.g. UCSC)
Provide a link to an anonymized genome browser session for "Initial submission" and "Revised version" documents only, to enable peer review. Write "no longer applicable" for "Final submission" documents.

Methodology

Replicates
Describe the experimental replicates, specifying number, type and replicate agreement.

Sequencing depth
Describe the sequencing depth for each experiment, providing the total number of reads, uniquely mapped reads, length of reads and whether they were paired- or single-end.

Antibodies
Describe the antibodies used for the ChIP-seq experiments; as applicable, provide supplier name, catalog number, clone name, and lot number.

Peak calling parameters
Specify the command line program and parameters used for read mapping and peak calling, including the ChIP, control and index files used.

Data quality
Describe the methods used to ensure data quality in full detail, including how many peaks are at FDR 5% and above 5-fold enrichment.

Software
KEGG profiling was used to perform pathway mapping and determine significantly enriched pathways using hypergeometric tests ChIP-seq datasets for murine REV-ERBα and REV-ERBβ obtained from Gene Expression Omnibus were analysed using the HOMER tool. BedGraphs are visualised using the Integrative Genomics Viewer (IGV) tool.
